# Recent Progresses in Stable Isotope Analysis of Cellulose Extracted from Tree Rings

**DOI:** 10.3390/plants10122743

**Published:** 2021-12-13

**Authors:** Silviu-Laurentiu Badea, Oana Romina Botoran, Roxana Elena Ionete

**Affiliations:** National Research and Development Institute for Cryogenics and Isotopic Technologies—ICSI Rm. Valcea, 4th Uzinei Street, 240050 Râmnicu Vâlcea, Romania; silviu.badea@icsi.ro (S.-L.B.); roxana.ionete@icsi.ro (R.E.I.)

**Keywords:** tree rings, stable isotopes, cellulose, climate change

## Abstract

In this work, the challenges and progression in stable isotope investigation, from the analytical tools and technical sample preparation procedures to the dendroclimatological experiments, were reviewed in terms of their use to assess tree physiological responses to environmental changes. Since the isotope signature of whole wood is not always a reliable tool in studying the climate changes, cellulose is often preferred as the study material in paleoclimatic studies. Nevertheless, the isotope analysis of cellulose is challenging due to the difficulty to remove the other wood components (extractives, lignin, pectin, and hemicelluloses). Additionally, in the case of hydrogen isotope analysis, about 30% of the hydrogen atoms of cellulose are exchanged with the surrounding water, which complicates the isotope analysis. In recent years, more automated isotope analysis methods were developed based on high temperature pyrolysis of cellulose, followed by the chromatographic separation of H_2_ from CO and by their individual isotope analysis using isotope ratio mass spectrometry. When used to investigate climate factors, the combined isotope analysis δ^13^C and δ^18^O appears to be the most promising isotope tool. In contrast, the role of δ^2^H values is yet to be elucidated, together with the development of new methods for hydrogen isotope analysis.

## 1. Introduction

Ecosystems and biodiversity are already being influenced by climate change, as well as a wide range of physical and biological processes in many regions of the world, with available observational evidence being indicated in the forest biomes [1]. These impacts can put stress on the forest ecosystem and alter its functioning and biodiversity, affecting the overall provision of forest ecosystem goods and services, such as wood production, carbon sequestration, and the benefits from forest recreation and passive use, and, ultimately, human well-being [2]. In this regard, when addressing the most likely consequences of climate change on forest ecosystem biochemical processes, it is critical to consider the interaction between all three compartments of terrestrial ecosystems: atmosphere (climate), biosphere (vegetation), and lithosphere (soil). Changes in any of these compartments will have an impact on the others, due to the net movement of mass and energy and the established mean residence periods among compartments [3]. In the current context, the understanding of climatic phenomena becomes a necessity; their natural cyclicity being able to provide valuable information to scientists exploring the causes and consequences of climate variability, contributing to the reconstructing of past atmosphere conditions. Furthermore, owing to the complex nature of climate, vegetation, and soil interactions, the response of forest populations to elevated CO_2_ concentrations can range considerably, requiring the use of accurate proxy records [4], such as isotopic markers.

**Figure 1:**
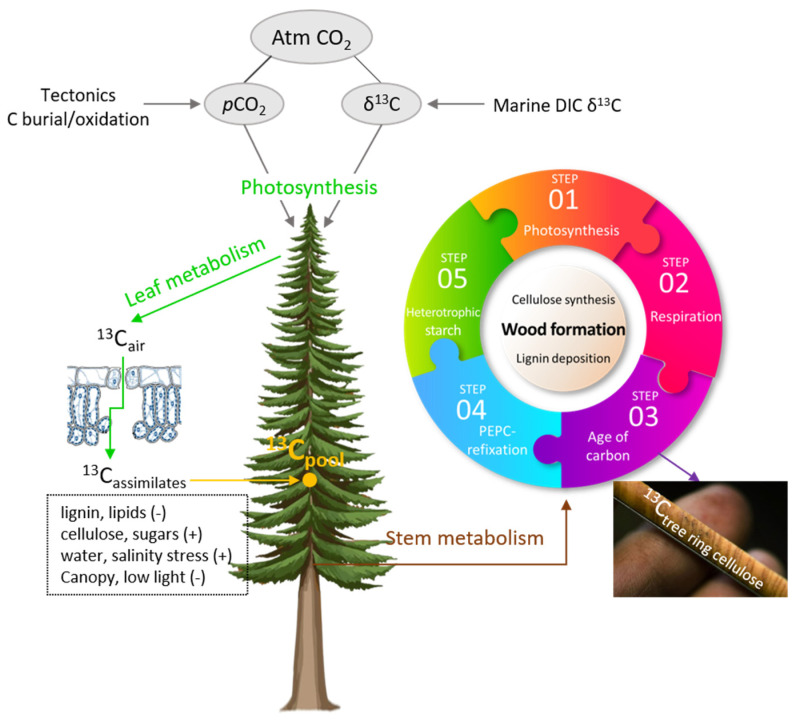
Photosynthetic and evaporative processes regarding the stable isotope composition of cellulose.

Stable isotopes are well-known integrators and tracers for a wide range of important physical and biological processes, providing time-integrated information regarding tree ecophysiological adaptations to varying abiotic factors. In the literature, several wood components (wood, lignin, or cellulose) were employed to investigate the stable isotopes of bioelements such as hydrogen (δ^2^H), carbon (δ^13^C), and oxygen (δ^18^O) [5,6,7,8,9]. In general, the δ^13^C in plant tissues offers a comprehensive record of the ratio of intercellular to atmospheric CO_2_ concentrations (see Figure 1) over the course of the carbon fixation stage [10].

Plant physiological stable isotope systems, when properly modelled, could effectively predict how δ^18^O and δ^2^H values of precipitation and climatic variables impact these values of the plant intrinsic water, as well as how they were registered into the plant’s organic elements. Considering that hydrogen, carbon, and oxygen stable isotope compositions reflect plant biogeochemical cycle, different wood components (wood, lignin, or cellulose) were implied in paleoclimatic studies. Nevertheless, due to the chemical complexity of wood (that contains cellulose, resins, lignins), the various components of wood can give divergent isotopic signatures [11]. Therefore, the isotope signature of whole wood is not always a reliable tool in studying the climate changes, due to the changing mass proportions of the different wood components (cellulose/lignin ratio) that can vary among the tree species and provide a wide range of biases. Since cellulose is a widespread primary carbohydrate, has a short synthesis pathway, unique chemical structure, but little mobility in tree rings, it is often the preferred test sample material for stable isotope analysis [12]. Cellulose has been traditionally divided in α-cellulose (fraction of cellulose that is insoluble in 17.5% NaOH solution), β-cellulose, and γ-cellulose. Many stable isotope studies of cellulose are referring to α-cellulose.

Compared with wood components produced in secondary metabolism of trees (e.g., lignin or fatty acids), the carbohydrates from primary metabolism of trees (i.e., sugars, starch, or cellulose) shown usually have heavier isotopic signatures [13]. With respect to ^13^C, and taking into consideration a non-statistical distribution of ^13^C in cellulose precursors, there is an enrichment of 2–4‰ of cellulose vs. lignin or fatty acids. Similarly, for ^18^O, the lignin is usually less enriched than cellulose, when taking into consideration non-statistical ^18^O distributions originating from individual metabolic reactions from trees [14]. For example, it was found that cellulose from white spruce wood was 10.5‰ ± 0.4 enriched in ^18^O than lignin [15].

Proportionally, cellulose and hemicelluloses (which together account for 65–75 percent of all wood) comprise the majority of the material. Considering this, the carbon isotope fingerprint of the whole wood is primarily provided by lignin, since cellulose and hemicelluloses have a lower absolute carbon content than lignin (by almost 50%). This change in the relative mass proportion between cellulose and lignin during tree growth (from pith to bark) influences the overall isotopic fingerprints of whole wood relatively to long-term records. Additional contribution of hydrogen, carbon, and oxygen isotopes might be given by extractives (alkaloids, polyphenols, fatty acids, essential oils, proteins, terpenes, pectins, waxes, resins, gums, starch, glycosides, saponins, etc.) which show a wide range of δ-values, since they are produced in the secondary plant metabolism [13]. However, they can have a minor impact on the total ^13^C value of wood, especially if these extractives only account for an insignificant fraction and/or their values are mostly in the cellulose and lignin isotope variability.

With respect to environmental and climate changes, some studies suggested that whole wood was more suitable to study the stable isotope chronologies, while others, on the contrary, argued that cellulose was the preferred sample material for stable isotope analysis [12]. Some studies hint that there are no statistical differences between δ^13^C and δ^18^O values of whole wood and cellulose, in relationship with climatic data [16]. In general, their observations are supported by a yet unaddressed hypotheses that in tropical, Mediterranean, and even cool temperate locations, excessive vapor pressure deficits during summer middays lowers the stomatal closure and hampers CO_2_ absorption, conducting to a decline of the photosynthetic rate that implies a reduction of sugar production. This then indicates that all daily conditions may not be registered in the isotopic composition of sugars and, therefore, cellulose. During the growth season, cellulose becomes a biased and incomplete recorder of the diurnal ambient circumstances if this process persists for several days. Considering that climate change is expressed as an increase in global temperature, this has the potential to intensify the photosynthetic midday depression, cause information loss in the cellulose isotopic composition, and lead to isotopic divergence, a phenomenon that has yet to be reported in the literature, according to some experts like Fu et al. [17]. According to the current trend, cellulose is a reliable archive for isotope-based climate reconstruction, if the yearly mean duration of the midday depression is brief and does not vary significantly over time. Conversely, other studies stated that the stable isotope analysis of cellulose is a necessary step in investigating the climate signals, since it is considered a better indicator of the past climate [18,19]. In a study, Schollaen et al. used the δ^13^C and δ^18^O values of non-extractives wood prepared from teak tree rings (*Tectona grandis*) to reveal dry and rainy season signals of rainfall in Indonesia [20].

Given the need for careful evaluations of stable isotopes as climate proxies for different regional environments and tree species, this overview of the up-to-date information on tree rings isotopic composition aims at: (1) describing the main extraction techniques and stable isotope analysis methods recently developed for the tree rings cellulose, and (2) reviewing research avenues to identify their correlation with climate changes.

## 2. Challenges in Development of Stable Isotope Analysis Methods for Cellulose

### 2.1. Sample Preparation

The ideal α-cellulose extraction procedure for isotopic measurements aims to remove extractives, lignin, pectin, and hemicelluloses from a wood sample, while preventing cellulose breakdown. For α-cellulose extraction from wood samples, there are currently over 10 different methods and procedures presented in the literature, including variants of the main three methods: (i) diglyme-HCl methods [21], (ii) Brendel type [22], and (iii) Jayme-Wise type [11,23,24] methods (see Table 1). Even with the simplest “bath” approach, cellulose extraction requires time; this procedure being particularly important and essential when investigating long-term chronologies for past climate reconstruction and for obtaining the strongest and most stable climatic signal. Over the last two decades, efforts have been made to reduce process time, improve cellulose separation simplicity, and evaluate the feasibility of various chemical procedures for the purification of cellulose from different plant types or tissues. Nevertheless, due to incomplete removal of extractives by delignification and alkaline hydrolysis used in the traditional chemical extraction methods [25], several pre-extraction methods with organic solvents were also developed [26].

#### 2.1.1. Pre-Extraction Methods

The pre-extraction methods consisted mostly in removal of extractives from wood with various organic solvents (ethanol, acetone, toluene, etc.), particularly used for conifers trees wood [14,29]. Wood extractives generally contain monomers, dimers, and polymers such as lipids, fatty acids, and alcohols. Among these, phenols, terpenes, steroids, and resin acids are present, as well as wax and tannic acids found in small amounts in these wood materials. Environmental stress events (such as fire or drought) can often be associated to extractives; these extreme conditions can either induce resin biosynthesis or function as a defence mechanism against microbial and/or herbivore attacks [11]. Changes in the relative proportions of wood components caused by environmental factors may enrich or deplete the isotopic signature predicted by fractionation models [31]. The solvent, or even only boiled water extraction procedures, usually provides a higher accessibility to lignin oxidation. By applying a pre-extraction method, Richard et al. observed a mass loss of 6% for pine and poplar, 12% for beech, and up to 15% for oak, but regarding the isotopic composition only the δ^13^C value of the pine increased significantly [32]. Furthermore, Harlow et al. [33] found a comparable high ^13^C offset between bulk wood and extractives free wood in 44 tree species. By using a continuous flow isotope ratio mass spectrometry (CF-IRMS) technique that provided precision better than ±0.2‰, recent work has shown that organic solvent extraction was unnecessary for the purification of α-cellulose from resinous pine wood samples. However, it is questionable if this is a general principle for α-cellulose processing of plant samples of any kind, since the quantity and structure of extractives in tree rings developed in other years, could be different. These extractives chemical behaviour after delignification and alkaline hydrolysis is still unclear. Since the interannual variation of tree ring isotope composition can be as high as the isotope analytical uncertainty, a test with a long and consecutive tree ring series, as well as a different isotopic technique, with a precision better than ±0.05‰ is required. However, for conifers, such a ^13^C offset is not always identified [34], implying that the extractives components removed have changed in proportion or their isotopic composition.

#### 2.1.2. Chemical Extraction Methods

For the preparation of the α-cellulose from wood for isotope analysis, each of the main chemical extraction methods mentioned above has advantages and disadvantages. The diglyme-HCl is one of the simplest methods for producing cellulose from whole wood and involves a single processing step that removes most extractives, hemi-cellulose, and lignin by using a mixture of diglyme (diethylene glycol dimethyl ether) and concentrated HCl solution under heating (i.e., 90 °C). Nevertheless, diglyme-HCl is not always a reliable extraction method when dealing with wood with high resin and lignin contents.

The Brendel method mainly involve the removal of lignin from whole wood with acetic and nitric acids, followed by the extraction of α-cellulose with ethanol and acetone [29,35]. Nevertheless, the main disadvantage of the Brendel method is the variability of purity with the tree species for the extracted α-cellulose [22]. As a good alternative to the above-mentioned two methods classes, the Jayme-Wise method consists of three major steps: (i) the use of acidified sodium chlorite solution for delignification (called chlorination), (ii) treatment and hydrolysis with a NaOH solution 17% (called purification), and (iii) treatment with HCl solution (neutralisation) [24]. The Jayme-Wise method applied in nine European laboratories by Boettger et al. have shown very accurate δ values for both ^13^C and ^18^O [36] that are within the precision of isotope ratio mass spectrometry measurements (±0.2‰ for δ^13^C and ±0.3‰ for δ^18^O).

### 2.2. Challenges in Developing EA-IRMS Methods for Hydrogen, Carbon, and Oxygen Isotopes

#### 2.2.1. Hydrogen Isotope Analysis of Cellulose

**Figure 2:**
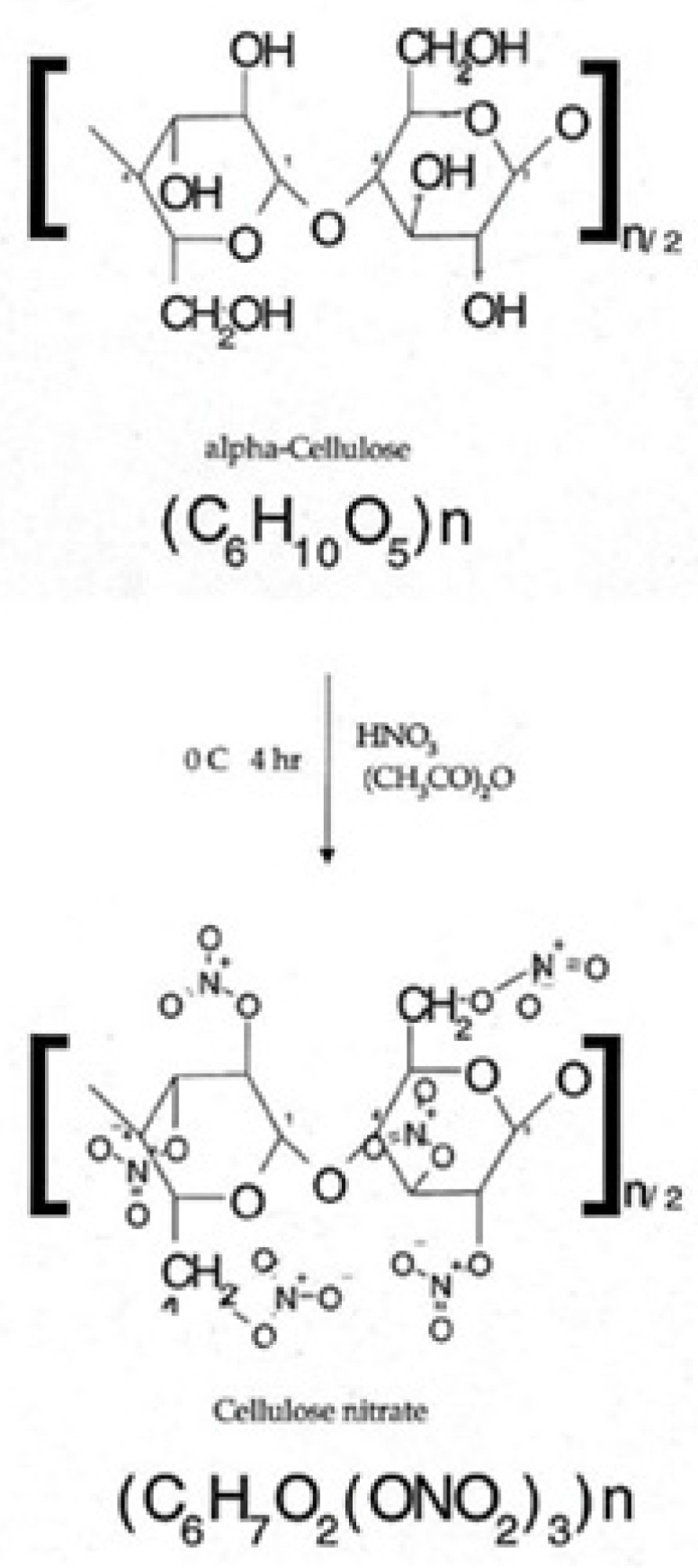
The nitration of α-cellulose for ^2^H analysis in the presence of acetic anhydride.

One of the first approaches used to measure the δ^2^H values of α-cellulose was to determine the D/H ratios of nonexchangeable hydrogen in cellulose by nitration of cellulose-to-cellulose nitrate (Figure 2) in various ways, followed by the combustion of cellulose nitrate in O_2_ excess and the cryogenic separation of H_2_O, as well as its reduction to hydrogen over hot uranium, while the δ^2^H values were measured using an isotope ratio mass spectrometer (IRMS) [37]. Another approach used to measure the δ^2^H values of α-cellulose is represented by purified cellulose equilibration with water of known isotopic composition, followed by combustion of cellulose at 900 °C for 3 h over CuO [38]. Afterwards, the CO_2_ and H_2_O were separated cryogenically, and the water was reduced to hydrogen over hot uranium; the δ^2^H values were measured using an IRMS. However, both methods present the disadvantage of measuring only the isotope ratio of nonexchangeable hydrogen from cellulose. In a recent alternative method, Nabeshima et al. (2018) determined the δ^2^H of whole cellulose extracted from *Quercus crispula* without nitrification of cellulose, using a continuous-flow system coupled to a pyrolysis elemental analyzer and an IRMS [39]. In this study, δ^2^H values reflect 30% of hydroxyl-H, which is exchangeable with that of water, especially during sodium hydroxide treatment for cellulose extraction, together with 70% of nonexchangeable hydrogen (carbon bound-H). Nevertheless, since all the cellulose samples were chemically extracted in a glass tube, it was demonstrated that D/H ratios for hydroxyl-H are uniform among all cellulose samples originating from the same tree [39], while assuming that the δ^2^H values of cellulose in a tree reflects proportionally that in the carbon bound-H. Other recent studies performed by Loader et al. (2014) [40] and Arosio et al. (2020) [5] highlighted a fast method to determine the δ^2^H of non-exchangeable hydrogen of α-cellulose extracted from the whole wood of different tree species by pyrolysis and continuous-flow isotope analysis which enabled the online equilibration of exchangeable hydrogen. A 1 m long and 5 Å molecular sieve heated at 50 °C was used for gas chromatography (GC) separation pyrolysis products, H_2_ and CO [40], while the δ^2^H values were determined by IRMS, using an IAEA-CH-7 PE polyethylene foil for two-point calibration of the mass spectrometer.

#### 2.2.2. Combustion of Cellulose for Carbon Isotope Analysis

Comparing with the determination of δ^2^H values of cellulose, for the δ^13^C values of α-cellulose extracted from whole wood appears to be a straight-forward procedure by using elemental analysis-isotope ratio mass spectrometry (EA-IRMS), the α-cellulose being combusted completely (at over 1000 °C) in an elemental analyser to CO_2_ and H_2_O [24,41]. The resulted CO_2_ was afterwards analysed by the IRMS, while various reference materials (i.e., L-Alanine IA-R041, Beet Sugar IA-R005, Cane Sugar IA-R006) could be used for calibration [42]. Nevertheless, it was observed that when using the Brendel method for α-cellulose extraction, the incomplete removal of lignin and resins may have led to an offset of δ^13^C values, with less than 1‰ more negative than the one of pure α-cellulose [22].

#### 2.2.3. Pyrolysis of Cellulose to Carbon Monoxide for Oxygen Isotope Analysis

**Figure 3:**
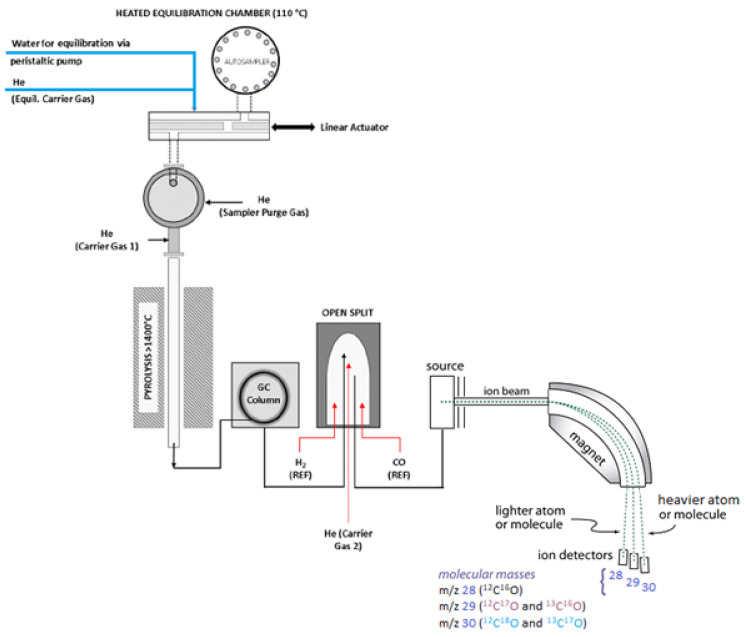
Analysis of hydrogen, carbon, and oxygen isotopic ratios in cellulose by thermal conversion elemental analysis (TC/EA) performed at high temperature (>1450 °C), coupled to GC column and an isotope ratio mass spectrometer (TC/EA-GC-IRMS). Adapted with permission from ref. [40]. Copyright 2014 American Chemical Society.

In contrast with δ^13^C, the δ^18^O values were determined through pyrolysis of α-cellulose to produce CO at high temperature (>1450 °C) in a Thermal Conversion Elemental Analyzer (TC/EA), coupled to an isotope ratio mass spectrometer [41]. The use of such high temperature reduction was suggested by Leuenberger and Filot [43], which showed in their study that fractionations associated with the reduction process carried out at temperatures above 1450 °C were minimal, approaching the stoichiometric expected value both for C and O. Nevertheless, in the last years, the new developed approaches trended towards simultaneous measurements of ^2^H, ^13^C, and ^18^O ratios from α-cellulose (see Figure 3), particularly for the last two isotopes [43]. The results of ^2^H/^1^H, ^13^C/^12^C, and ^18^O/^16^O isotope ratios measurements performed by Loader et al. (2014) [40] showed a precision comparable with that of other conventional techniques (δ^2^H 3.0‰, δ^18^O 0.30‰, δ^13^C 0.15‰). Furthermore, in a recent study performed by Andreu-Hayles et al. (2019), the δ^18^O and δ^13^C values of α-cellulose were determined by measuring the CO isotopologues (^12^C^16^O, ^13^C^16^O, ^12^C^18^O, ^12^C^17^O, and ^13^C^17^O) on three different IRMS collectors that recorded the masses 28, 29, and 30 (see Figure 3), with the obtained δ^13^C values agreeing with those determined using EA-IRMS [41].

## 3. Assessing the Isotope Ratios of Hydrogen, Carbon, and Oxygen in Cellulose Extracted from Tree Rings and Its Correlation with Climate Variables

### 3.1. Assessing the Isotope Ratios of Hydrogen in Tree Rings-Cellulose and Its Correlation with Climate Variables

The hydrogen isotope ratios of α-cellulose were not applied often to investigate the climate changes [44]. The above-mentioned analytical issues with exchangeable hydrogen (hydroxyl-H) in cellulose [38] made δ^2^H values difficult to be used as proxy for climate study. Nevertheless, recent studies indicated that multiple enzymatic reactions from plants, induced a significant isotope fractionation of ^2^H which resulted in plant carbohydrates, such as cellulose. When it comes to the isotope composition of ^2^H plant celluloses, three factors are involved: the water source’s isotope composition (in this case, δ^2^H value), leaf evaporation (which enriched heavier isotopes in the liquid phase), and biosynthetic isotopic fractionation (that included many complex biochemical pathways) [45]. The fractionation of hydrogen isotopes was linked to environmental and physiological factors together. The effect of the environment on transpiration and evaporation caused leaf water to evaporative ^2^H-enrichment in the plant, causing δ^2^H of cellulose to be affected by both stomatal conductance and the climate’s impact on transpiration [46]. A strong correlation among cellulose δ^18^O and δ^2^H could, therefore, reflect the source (i.e., water) and environmental impacts [47], while the absence of correlation might have indicated even a supplementary hydrogen [48] or oxygen [49] isotope fractionation impact. It was demonstrated that cellulose extracted from the leaf of deciduous tree species was enriched in deuterium during the early growth season, when the availability of fresh assimilates from photosynthesis was still minimal [50]. Furthermore, early wood was more ^2^H-enriched compared to late wood of the same year, which led to intra-annual variations in tree-rings [39]. However, further studies are needed to elucidate the role of δ^2^H values in response of different trees species to climate changes.

### 3.2. Assessing the Isotope Ratios of Carbon in Tree Rings-Cellulose and Its Correlation with Climate Variables

The δ^13^C values of tree-ring cellulose have been used systematically since the 1980s as indicators of past climates [51]. Factors such as atmospheric CO_2_ concentrations [52,53] and soil moisture [54] affected stomatal conductance, which significantly influenced the photosynthetic activity. Other factors affecting stomatal conductance included atmospheric vapour pressure deficit, as well as nutrient availability. Thus, δ^13^C was usually correlated with air humidity or precipitation in dry environments, whereas it was associated with irradiance factors and growing season temperature in humid environments [12]. Several metabolic events affected δ^13^C values of tree-ring cellulose related to the postcarboxylation fractionation, including the loading and transit of phloem and the respiratory isotope fractionation [55]. A study applied δ^13^C values of cellulose extracted from latewood of oak (*Quercus robur* L.) to investigate the climate variations in two sites from eastern England (UK), with different hydrological characteristics [56]. It was observed that the July and August environmental variables recorded at both sites correlated with the variance in the δ^13^C values recorded for the cellulose extracted from latewood. In another study, the δ^13^C values of cellulose extracted from annual tree ring of three species (*Fagus sylvatica* L., *Quercus robur* L., and *Pinus sylvestris* L.) were used to study the climate variations from Woburn Abbey, Bedfordshire, England [57]. It was found that high δ^13^C variations in the cellulose of all three species correlated significantly with average July–October maximum temperatures and with average June–September relative humidity, while all three species had similar responses to climate variations [57].

A correlation of δ^13^C values of cellulose extracted from the earlywood/latewood layers of eastern Siberian tree rings with the soil water conditions was performed [58]. The δ^13^C values of both cellulose and whole wood agreed with the summer climate (July and August) according to a study on cellulose and whole wood from tree ring of *Quercus robur* L. sampled from an area in eastern England, UK [7]. Additionally, δ^13^C together with δ^14^C obtained from the analysis of α-cellulose and whole wood extracted from annual rings of pine and oak from different sites from Poland were used to reconstruct climate changes during the last 400 years [59].

### 3.3. Assessing the Isotope Ratios of Oxygen in Tree Rings-Cellulose and Its Correlation with Climate Variables

The δ^18^O values of α-cellulose extracted from tree rings was used in the investigation of climates changes. The oxygen isotopic composition of tree ring cellulose is a crucial data source in much of this research direction. For example, although there are substantial uncertainties and information gaps, the hypothesis of what controls δ^18^O variance in tree-ring cellulose is quite well developed. Several numerical models can characterize the link between these variables and δ^18^O values from cellulose and could be used to explore oxygen isotope fluctuations in various scenarios [60]. Cellulose oxygen isotopes are influenced by a wide range of factors, including (i) the isotopic composition of the water source (which is determined by precipitation isotopes, soil residence time, and evaporative effects); (ii) leaf water enrichment as a result of transpiration [61]; (iii) fractionation between leaf water and carbonyl oxygen [48,62]; and (iv) other processes of oxygen exchange [60]. Tree-ring cellulose δ^18^O values can be used to reconstruct the oxygen isotopic composition of precipitation due to the intrinsic relationship with source water oxygen isotopes. As a result, cellulose δ^18^O values can be used to reconstruct global and regional hydrological variation, such as changes in basinal water regimes [63] or large-scale atmospheric circulation patterns [64]. For example, δ^18^O values of α-cellulose extracted from tree rings of four different types of forests (temperate forest, Central Massachusetts (USA), dry forest, Liberia, (north-western Costa Rica), rain forest, La Selva (eastern Costa Rica), dry forest, Piura (northwest coastal Peru)) were used by Evans and Schrag [65] to develop proxy chronological, rainfall, and growth rate estimates from tropical trees, with the aim to improve reconstructions of the past behavior of tropical climate phenomena over the past few hundred years [65]. It was found that the annual cycle of local rainfall and relative humidity for the temperate forests from US and Costa Rica were correlated with the δ^18^O values of α-cellulose that showed cyclic isotopic signatures of several permille (SMOW).

According to Battipaglia et al. [18], the δ^18^O of cellulose for two species of tree (*Fagus sylvatica* L. and *Acer pseudoplatanus* L.) originated from Monti Picentini (Italy) was well correlated with the monthly temperature during the growing season. For δ^18^O of whole wood this observation was not valid.

### 3.4. Multi-Element Isotope Analysis of Tree Rings-Cellulose

δ^13^C–δ^18^O correlation is usually used in the studies of climatic signals, as the dual isotope signatures of cellulose reveal better information when compared with individual isotopes. Porter et al. [66] used the δ^13^C and δ^18^O values of whole-ring α-cellulose to study the δ^13^C and δ^18^O variability in tree rings from three trees at a high-latitude tree line site in north-western Canada to reveal the climate signals from the period 1850–2003. Reynolds-Henne et al. [67] used δ^13^C and δ^18^O values of cellulose extracted from tree ring wood of oak and pine from Ticino, Switzerland, to investigate climate (precipitation amount and temperature) for the timeframe 1660–2000. They found that δ^18^O values were influenced by large-scale synoptic circulation and that the δ^13^C values reflected local climatic conditions, while both isotope signatures reflected conditions of the current growing season. Another observation was that correlations between δ^13^C and δ^18^O values of tree ring cellulose and the climate were temporally unstable, apart from δ^13^C of cellulose extracted from pine, while the oak showed the highest climate signals for the 20th century tree rings. More recently, Mischel et al. [29] analysed α-cellulose, as well as whole wood sampled from *Pinus sylvestris* L. from the Altenkirchen region (Germany) and used the δ^13^C and δ^18^O values as climate proxy. Mischel et al. [29] found that the water balance and annual evapotranspiration were correlated with δ^18^O values of α-cellulose and whole wood, while the summer temperatures and growing season precipitation were found to be correlated with δ^13^C values. Nagavciuc et al. used δ^13^C and δ^18^O values of cellulose extracted from the tree ring of *Pinus cembra* L. in the region of the Călimani Mountains (Romania) to elucidate the isotopic chronologies of 1876–2012 [68]. They observed a significant correlation of δ^18^O values with the maximum temperature values, local relative humidity, and cloud cover, while δ^13^C signatures correlated with the local April–May relative humidity and regional summer precipitation.

**Figure 4:**
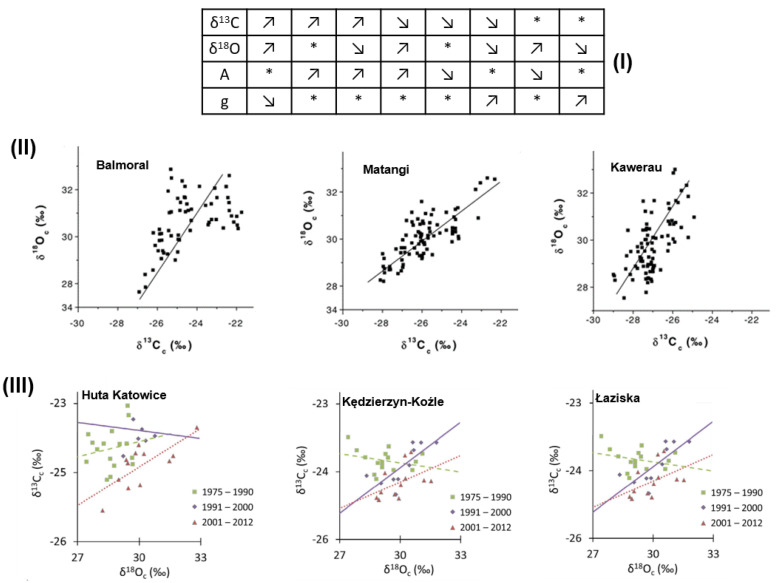
(**I**) Theoretical scenarios [71] for the interaction between the stomatal conductance (**g**) and photosynthesis (**A**) and isotope composition of plants (up- or downward arrows represent increasing or decreasing values, * indicates insignificant changes; (**II**) Relationship between δ^13^C and δ^18^O of cellulose extracted from two annual tree rings of *Pinus radiata* at three different sites from New Zealand: Balmoral (δ^18^O = 69.1 + 1.57·δ^13^C; r = 0.52), Matangi (δ^18^O = 54.6 + 0.95 × δ^13^C; r = 0.77), and Kawerau (δ^18^O = 88.5 + 2.17 × δ^13^C; r = 0.63); (**III**) Comparison of the δ^13^C and δ^18^O series for the investigated sites: Huta Katowice (1975–1990: δ^13^C = 0.11 − 27.1 × δ^18^O, r = 0.18; 1991–2000: δ^13^C = −0.058 − 21.8 × δ^18^O, r = 0.37; 2001–2012: δ^13^C = 0.28 − 32.7 × δ^18^O, r = 0.74), Kędzierzyn-Kożle (1975–1990: δ^13^C = −0.068 − 21.8 × δ^18^O, r = 0.28; 1991–2000: δ^13^C = −0.34 − 34.1 × δ^18^O, r = 0.77; 2001–2012: δ^13^C = 0.20 − 30.1 × δ^18^O, r = 0.47), and Łaziska (1975–1990: δ^13^C = 0.38 − 34.8 × δ^18^O, r = 0.83; 1991–2000: δ^13^C = −0.31 − 33.2 × δ^18^O, r = 0.82; 2001–2012: δ^13^C = −0.027 − 23.1 × δ^18^O, r = 0.064) for the three periods of time (**III**). Reprinted with permission from refs. [69,74].

Barbour et al. [69] investigated the relationship between δ^18^O and δ^13^C of cellulose extracted from two annual tree rings of *Pinus radiata* at three different sites from New Zealand (Balmoral, Matangi, and Kawerau) using the δ^18^O–δ^13^C plot (Figure 4I). They found that although the slopes were different, the δ^13^C and δ^18^O values were positively correlated at all three sites. The highest correlation coefficient was found at the Matangi site, followed by the Kawerau and Balmoral site. Regarding the slope values, they varied from +0.95‰ δ^18^O and 1‰ δ^13^C (Matangi site), to 1.57 δ^18^O per 1‰ δ^13^C (Balmoral site), with the highest being recorded at the warmest Kawerau site (2.17 δ^18^O per 1‰ δ^13^C). The results found by Barbour et al. [69] corresponded with the previous findings of Saurer et al. [70], stating that a higher oxygen isotope enrichment in the leaf water (thus higher δ^18^O values) at the dry and warm sites were attributable to the enhanced stomatal resistance (thus higher δ^13^C values). A more interesting approach was to use the combined analysis of carbon and oxygen isotopes (δ^13^C–δ^18^O plot) of tree rings cellulose to indicate the proportion of stomatal conductance and photosynthesis to the variation of intrinsic water use efficiency (WUE_i_) in response to elevated CO_2_ levels [71,72] (Figure 4II). This ‘dual-isotope’ approach considered the interpretation of δ^13^C variation by measuring oxygen isotopic composition (δ^18^O) of the same material, since if changes in environmental conditions caused a long-term change in carbon assimilation (A, demand for CO_2_) and/or stomatal conductance (g, supply of CO_2_), this then should have been reflected in the carbon and oxygen isotope ratios of organic matter [73].

In contrast with Barbour et al. [69], the study performed by Sensuła et al. [74] in 2018 used the reversed δ^13^C–δ^18^O plot to study the relationship between tree-ring cellulose δ^13^C and δ^18^O and the climate over the period 1975–2012 for Scots pine from an industrial zone of Poland. Their study showed that the δ^13^C–δ^18^O linear correlation changed from the period of 1957–1990, prior to significant modernisation in the factories, to the period of 1991–2000, when there was a significant modernisation in the factories from the same industrial area. Applying the model developed by Scheidegger et al. [71], Sensuła et al. [74] found positive correlations δ^13^C–δ^18^O for certain periods of time. For example, the highest positive correlation δ^13^C–δ^18^O for the Kędzierzyn-Kożle region was found between 1991–2000. For the Łaziska region, the highest correlations (Figure 4III)) were found between 1975–1990 and 1991–2000, while for the Huta Katowice region, the highest correlation was found in the period 2001–2012.

### 3.5. Assessing the Preservation of Isotope Ratios of Hydrogen, Carbon, and Oxygen in Cellulose during Wood Decay

The isotope signatures of the decayed wood and cellulose extracted from the decayed wood can be used to assess the mechanisms of tree mortality associated with climate changes. English et al. [75] investigated the δ^13^C and δ^18^O values of fresh and decayed whole sapwood and corresponding extracted α-cellulose obtained from four tree species (*Pinus ponderosa*, *Juniperous monosperma*, *Pinus edulis*, and *Abies concolor*). They found no difference between live and decayed trees, with respect to offset between δ^13^C and δ^18^O signatures of wood and α-cellulose obtained across a wide range of inter-annual and regional climate differences. Savard et al. [76] used δ^13^C and δ^18^O values of decayed whole, cellulose, and lignin extracted from tree rings of *Picea mariana* which showed different degrees of wood textural preservation and were obtained over the last millennium. They found a decrease in δ^18^O values of cellulose, while the δ^13^C of cellulose were preserved.

Nagavciuc et al. [30] performed a comparative study of δ^13^C and δ^18^O values of cellulose extracted from decayed and non-decayed wood samples of Swiss stone pine (*Pinus cembra*) trees. Values between 0.5 and 1.6‰ were recorded for δ^13^C values of cellulose extracted from intra-rings (around the circumference of a single ring) and between 2 and 4.7‰ for inter-trees (non-decayed wood). Simultaneously, the δ^18^O values were 0.1 and 0.5‰ for intra-ring cellulose and 1.1 and 2.3‰ for inter-tree. The study concluded that the decayed wood could be used for isotopic paleoclimate research, since the differences of δ^18^O and δ^13^C values between decayed and non-decayed wood were smaller than the variation among different trees from the same site.

## 4. Research Trends Regarding Isotope Ratios of Hydrogen, Carbon, and Oxygen in Tree Rings

**Figure 5:**
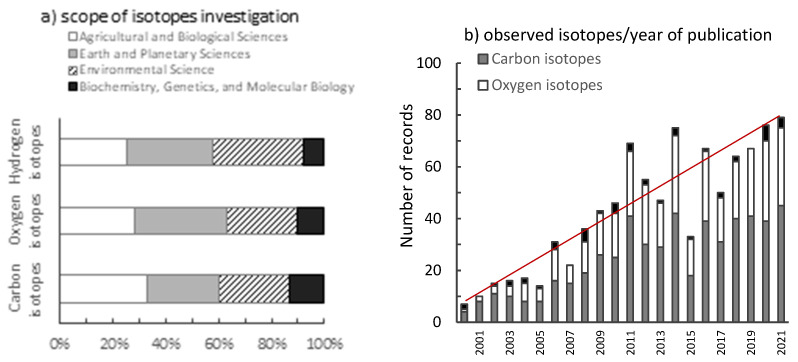
Search results for (“tree rings” AND “stable isotopes”) in the title, abstract, or keywords of scientific literature that were produced from a search for the subject area’s scholarly output that was published. Even though the first study on the subject was published in 1954 [77], we focused on representing the fact that the interest in the area has risen dramatically since the 2000s. (**a**) scope of the study; (**b**) investigated element. Source: Scopus (accessed on 11 September 2021).

The greater understanding of the underlying physiological and biochemical processes responsible for wood isotopic ratios, as observed, has undoubtedly resulted in increased usage of wood isotopic fingerprints throughout terrestrial biomes and in a variety of associated fields in recent years. A clear demonstration of this is supported by the temporal distribution and exponential growth of the total number of research articles recovered from the Scopus database that matched the keywords “tree rings” AND “stable isotopes”, summing up about 550 papers for ^13^C, 348 for ^18^O, and 46 for ^2^H, for works published up until November 2021 and that were published in peer-reviewed journals (Figure 5). Among the most common subject area of stable isotopes in tree rings are forestry and ecology (which account for 45% of published studies), climatology (approximately 34%), and other fields such as archaeology or hydrology (22%).

More than 85% of stable isotopes pattern in tree rings papers were represented by original research articles published in peer-reviewed journals, with review articles and conference proceedings accounting for 12%, and book chapters/books accounting for 3%. Hydroclimate proxies’ variation, age trends, abiotic factor influence on tree resistance, recovery, resilience and growth, and different tree species physiology were all important facets of this domain. A trend depicted by the increase in popularity during the last four years was that stable isotope techniques were emerging in climate change, which currently represent a top subject. Stable isotopes models maintain the well-known stability of large-scale geographical patterns of change over a transient experiment, supporting pattern scaling to reflect changes across time and different situations.

## 5. Conclusions

This review is presenting a comprehensive study on isotopic investigation of cellulose extracted from tree rings and its potential application in elucidating climate changes. While the role of δ^13^C and δ^18^O values in investigating the climate changes is relatively established, the role of δ^2^H values is yet to be elucidated. Observing that tree ring components have distinct isotopic ratios, most studies have focused on single components, most commonly cellulose, and isotope proxies (usually carbon or oxygen). The isotope fingerprint of cellulose is typically regarded to be the most relevant marker of environmental knowledge during tree growth, since there is no material transfer and no additional fractionation. Since the physiological process that leads to fractionation differs significantly between isotopes, a multi-proxy strategy based on the three primary isotopes and a variety of components appears to be the most effective method for enhancing climate reconstruction research.

## Figures and Tables

**Table 1:** Methods for cellulose extraction from wood.

No.	Extraction Method	Tree Species	δ^13^C [‰] Cellulose	δ^18^O [‰] Cellulose	Ref.
**Pre-Extraction Methods**					
1	Organic solvent pre-extraction of α-cellulose was processed in a Soxhlet system for 6 h with a 2:1 mixture of benzene to methanol, and another 6 h in acetone. Afterwards, extraction with acetic acid-acidified sodium chlorite solution followed by alkaline hydrolysis (modified Jayme-Wise method)	*Pinus koraiensis*	From −23.09 to −24.63‰ using solvent extraction for α-cellulose. From −23.38 to −24.89‰ without using solvent extraction for α-cellulose	-	[26]
2	Pre-treatment with 300 + 200 mL acetone, 200 mL mixed solvent (100 mL toluene and 100 mL ethanol) and again 200 mL acetone	*Peronema canescens Jack*	-	Average values around +19‰ upon solvent extraction	[27]
3	Pre-treatment in a Soxhlet extractor using a 2:1 mixture of toluene and denatured alcohol, with 8 h of refluxing. Additionally, an acetone pretreatment that was completed by an overnight soaking in deionized water, followed by an 8-day-soaking in acetone (acetone was replaced every 2 days)	*Loblolly pine* (*Pinus taeda* L.), *Norway spruce* (*Picea abies* (L.) *Karst.*), *Fraser fir* (*Abies fraseri* (*Pursh*) *Poir*.), *Ponderosa pine* (*Pinus ponderosa* D.), *Douglas fir* (*Pseudotsuga menziesii* (*Mirb*.) *Franco*), *Black spruce* (*Picea mariana Mill*.)	For *Loblolly pine* (GA, USA) in dry year: −24.25 ± 0.02‰ (with acetone pretreatment), respectively −24.36 ± 0.02‰ (with traditional pretreatment) For *Loblolly pine* (GA, USA) in rainy year: −26.74 ± 0.01‰ (with acetone pretreatment), respectively −26.79 ± 0.01‰ (with traditional pretreatment) For *Ponderosa pine*: −22.96 ± 0.02‰ (with pretreatment with acetone), respectively −22.99 ± 0.01‰ (with traditional pretreatment) For *Black spruce*: −23.88 ± 0.03‰ (with acetone pretreatment), respectively −23.83 ± 0.15‰ (with traditional pretreatment) For *Douglas fir*: −22.93 ± 0.02‰ (with acetone pretreatment), respectively −22.87 ± 0.02 (with traditional pretreatment) For *Norway spruce*: −26.67 ± 0.02‰ (with pretreatment with acetone), respectively −26.65 ± 0.00‰ (with traditional pretreatment) For Fraser fir: −24.26 ± 0.15‰ (with pretreatment with acetone), respectively −24.22 ± 0.06‰ (with traditional pretreatment)	For *Loblolly pine* (GA, USA) in dry year: +32.39 ± 0.06‰ (with acetone pretreatment), respectively +32.34 ± 0.07‰ (with traditional pretreatment) For *Loblolly pine* (GA, USA) in rainy year: +30.01 ± 0.15‰ (with acetone pretreatment), respectively +30.14 ± 0.21‰ (with traditional pretreatment) For *Ponderosa pine*: +30.77 ± 0.12‰ (pretreatment with acetone), respectively +30.48 ± 0.15‰ (traditional pretreatment) For *Black spruce*: +25.03 ± 0.10‰ (with acetone pretreatment), respectively +25.16 ± 0.02‰ (with traditional pretreatment) For *Douglas fir*: +30.25 ± 0.17‰ (with acetone pretreatment), respectively +29.93 ± 0.11 (with traditional pretreatment) For *Fraser fir*: +27.94 ± 0.34‰ (with pretreatment with acetone), respectively +27.20 ± 0.27‰ (with traditional pretreatment)	[28]
**Chemical Extraction Methods**					
4	Modified Brendel method: hydrolysis with acetic and nitric acid, followed by extraction with ethanol and acetone	*Pinus sylvestris*	From −24.03 to −22.66‰	From +32.73 to +34.33‰	[29]
5	Dyglime-HCl method	*E. maculata*, *E. botryoides*, *E. resinifera*, *P. pinaster* *C. glaucophylla* (wood)	From −26.0 to −27.0‰ (*E. maculate*) From −25.9 to −26.8 (*E. botryoides*) From −25.9 to −26.7‰ (*E. resinifera*), From −22.6 to −24.3‰ (*P. pinaster)*, From −21.5 to −22.9‰ (*C. glaucophylla)*.	From +29.7 to +31.8‰ (*E. maculate*) From +27.7 to +30.4‰ (*E. botryoides*) From +29.7 to +30.1‰ (*E. resinifera*), From +31.5 to +35.0‰ (*P. pinaster)*, From +28.1 to +34.0‰ (*C. glaucophylla)*.	[21]
6	Jayme-Wise method with toluene/ethanol extraction, bleaching with NaClO_2_, and purification with NaOH	*Pinus cembra*	Intra-ring carbon stable isotope variability between 0.5 and 1.6‰ Inter-tree between 2 and 4.7‰	Intra-ring oxygen stable isotope variability between 0.1 and 0.5‰ Inter-tree between 1.1 and 2.3‰	[30]

## Data Availability

All data included in the main text.
